# Dysentery—Mortality of Chicago for July

**Published:** 1857-08

**Authors:** 


*Dysentery—Mortality of Chicago for July.*

We take the following statistics from the *Daily Democratic Press.* It will be seen that the number of deaths for July is not above the average for the last three or four years; but it will be noted that an unusually large proportion of them are attributed to dysentery, and that more than two-thirds of the whole are children under three years of age. We have time to make but one remark on these results. We are satisfied that three-fourths of all this infant mortality is unnecessary. Scarcely a day passes that we do not see from a dozen to twenty of these sick children, and in regard to two-thirds of them the story is the same. The mother presents her baby, generally between three and twenty months old, emaciated to a skeleton, the skin hanging in wrinkles around its neck and limbs, its lips thin and pale, eyes sunken with that peculiar expression of meek sadness on its countenance which is so characteristic of this form of disease; and when asked how long the child has been sick, she answers, "Oh, it has had some diarrhœa two, three, and sometimes four weeks; but it was teething, and we did not think it much sick until the last few days, the discharges have become very bad, looking like *corruption*, and often mixed with blood." "But why did you not do something for the child sooner?" "Because it was *cutting teeth* that caused the difficulty, and we did give it *castor oil* every few days." Now it is precisely this exceedingly erroneous idea, that because a child is "cutting its teeth," its diarrhœa is harmless, or needs no checking, which causes a sacrifice of more than one hundred infants in Chicago every July and August, simply because, under the influence of the error, a disease easily controlled in its early stages is allowed to go on, and often aggravated by castor oil or vermifuges, until the lining membrane of the bowels is ulcerated, the mesenteric glands enlarged, and the child is fatally exhausted. It is high time that the profession should communicate better information to all mothers with whom they come in contact.

The following are the statistics, viz.;

MORTALITY OF THE CITY.

The total mortality for July is 250, which is a decrease, compared with the corresponding month last year.

The mortality for July for a series of years has been as follows:

| '47 | '48 | '49 | '50 | '51 | '52 | '53 | '54 | '55 | '56 | '57 |
|---|---|---|---|---|---|---|---|---|---|---|
| 58 | 41 | 411 | 240 | 67 | 179 | 111 | 934 | 136 | 266 | 250 |

The diseases of the deceased of July, 1857, were as follows: consumption, 12; unknown, 1; dysentery, 92; accident, 7; jaundice, 1; measles, 17; brain fever, 1; inflammation of the lungs, 2; teething, 37; croup, 14; drowned, 5; scrofula, 2; scarlet fever, 5; whooping cough, 1; congestive chills, 1; typhoid fever, 4; typhus fever, 4; tumor of groin, 1; cholera infantum, 5; convulsions, 1; water on brain, 1; delirium tremens, 3; puerperal fever, 1; still born, 6; bilious fever, 2; diarrhœa, 15; coup de soleil, 1; inflammation of the bowels, 2; dropsy, 1; marasmus, 1; old age, 1. Total, 250.

Of the number of deaths for the past month, 197 were children under ten years of age, most of them under 3 years. Of the 92 deaths by dysentery, 74 were children under 6 years of age, most of them from 1 to 2.

The nativities of the deceased of the past month are as follows: Chicago, 180; other parts of the United States, 7; Ireland, 32; Germany, 11; Norway, 3; France, 1; Prussia, 1; Wales, 1; unknown, 14.

